# Supplementary material for: Spatial Transcriptomic Profiling Reveals Microenvironment-Dependent Immune Signatures in a Lyme Arthritis model
Source: bioRxiv. 2026 Jun 25:2026.06.25.734510. Preprint. [Version 1] doi: 10.64898/2026.06.25.734510 (PMC13320799; doi:10.64898/2026.06.25.734510)
Supplement: 1 [file NIHPP2026.06.25.734510v1-supplement-1.pdf]

bioRxiv preprint doi: https://doi.org/10.64898/2026.06.25.734510; this version posted June 25, 2026. The copyright holder for this preprint (which was not certified by peer review) is the author/funder, who has granted bioRxiv a license to display the preprint in perpetuity. It is made available under a CC-BY 4.0 International license.

## SUPPLEMENTARY FIGURES

bioRxiv preprint doi: https://doi.org/10.64898/2026.06.25.734510; this version posted June 25, 2026. The copyright holder for this preprint (which was not certified by peer review) is the author/funder, who has granted bioRxiv a license to display the preprint in perpetuity. It is made available under a CC-BY 4.0 International license.

**Supplementary Figure 1. Selection of Regions of Interest and location of probes in *Borrelia burgdorferi*-infected joint tissue.**

(A) Representative immunofluorescence images of ankle joint sections from uninfected and *Bb*-infected mice at weeks 2 and 4 post-infection. Circles show regions of interest (ROIs) selected for spatial transcriptomics, each circle contained approximated 100 cells. Tissue sections were stained with SYTO-13 (blue; nucleic acid counterstain), anti-I-E$^k$ (red; MHC class II marker), and anti-Vimentin (VIM; yellow). Week 2 Uninfected, Week 2 Infected, Week 4 Uninfected, and Week 4 Infected are shown. (B) Table with probe numbers for each tissue.

**Supplementary Figure 2. Pathway analysis for downregulated genes following 2 and 4 weeks of *Bb* infection.** Top enriched MSigDB Hallmark pathways (A) and Metabolomics metabolites (B) in the downregulated genes at week 2 (top two graphs) and week 4 (bottom two graphs) after *Bb* infection. X axis indicates –log10(p-value) respectively were determined using EnrichR and Appyter. Blue bars are more significant, while gray bars have less significance.

**Supplementary Figure 3. Gating strategy.**

Unstained controls were used to set gates for each fluorochrome, and single-stained controls were used to calculate compensation. The hierarchical gating strategy for the identification of double-positive cells expressing the adhesion molecules CD106 (VCAM-1) and CD54 (ICAM-1) within the CD29+CD90.2+ synovial stromal cell population is illustrated with representative plots from Vehicle and *Bb* groups at week 2 and week 4

bioRxiv preprint doi: https://doi.org/10.64898/2026.06.25.734510; this version posted June 25, 2026. The copyright holder for this preprint (which was not certified by peer review) is the author/funder, who has granted bioRxiv a license to display the preprint in perpetuity. It is made available under a CC-BY 4.0 International license.

post infection (panels A-I). At least 100,000 were collected per sample. Prior to downstream gating, data quality was assessed using the FlowAI plugin (FlowJo v10.10.1) to automatically detect and quantify anomalous events based on default quality control metrics (flow rate, signal acquisition and dynamic range). A representative histogram (A) and time (x-axis) versus forward scatter-height (FSC-H, y-axis) plot (B) illustrate the distribution of “good events” (retained for further analysis) and bad events (flagged/anomalous) events. Singlets were then gated from good events on an FSC-area (FSC-A) versus FSC-H plot to exclude doublets and aggregates (C). The singlet population was then gated on FSC-A versus side scatter-area (SSC-A) to remove residual debris and select cells for further analysis (D). Viable cells were selected as cells negative for the viability dye (Fixable Viability Dye APC-Cy7) within the FSC-A/SSC-A gated cells (E). CD45 expression versus SSC-A on live cells was used to discriminate against hematopoietic lineages (CD45+) populations (F). The CD45- subpopulation was gated for further gating of CD31- events to remove endothelial contaminants (G). Synovial stromal cells were identified as CD29 (Integrin-b1) and CD90.2 (Thy-1.2) double-positive cells within CD45-CD31- population (H). Cells double positive for the adhesion molecules CD106 (VCAM-1) and CD54 (ICAM-1) were identified within the CD29+CD90.2+ synovial stromal cell gate (I). The flow cytometry results were analyzed using FlowJo Software v10.10.1 (Becton, Dickinson and Company).

**Supplementary Figure 4. Pathway analysis for upregulated genes in lateral synovium after 2 weeks of Bb infection**. Top enriched MSigDB Hallmark pathways (A) and Metabolomics metabolites (B) in the upregulated genes at week 2 after Borrelia

bioRxiv preprint doi: https://doi.org/10.64898/2026.06.25.734510; this version posted June 25, 2026. The copyright holder for this preprint (which was not certified by peer review) is the author/funder, who has granted bioRxiv a license to display the preprint in perpetuity. It is made available under a CC-BY 4.0 International license.

infection. X axis indicates –log10(p-value) respectively were determined using EnrichR and Appyter. Blue bars are more significant, while gray bars have less significance.

bioRxiv preprint doi: https://doi.org/10.64898/2026.06.25.734510; this version posted June 25, 2026. The copyright holder for this preprint (which was not certified by peer review) is the author/funder, who has granted bioRxiv a license to display the preprint in perpetuity. It is made available under a CC-BY 4.0 International license.

Gura et al,
Supplementary Figure 1

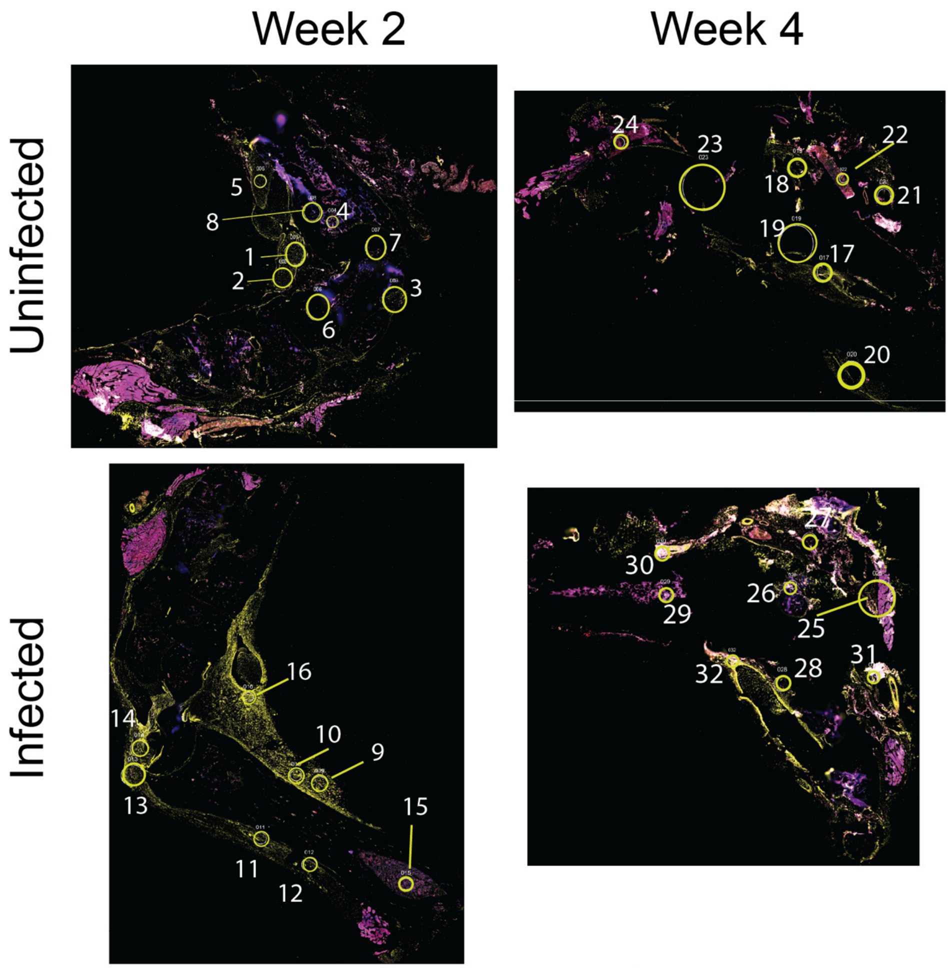


Probes

| | Week 2 | Week 4 |
|---|---|---|
| Uninfected | 25, 26, 27, 28, 29, 30, 31, 32 | 17,18,19,20,21,22,23,24 |
| Infected | 9,10,11,12,13,14,15,16 | 1,2,3,4,5,6,7,8 |

bioRxiv preprint doi: https://doi.org/10.64898/2026.06.25.734510; this version posted June 25, 2026. The copyright holder for this preprint (which was not certified by peer review) is the author/funder, who has granted bioRxiv a license to display the preprint in perpetuity. It is made available under a CC-BY 4.0 International license.

**Supplementary Figure 1. Selection of Regions of Interest and location of probes in *Borrelia burgdorferi*-infected joint tissue.**

**(A)** Representative immunofluorescence images of ankle joint sections from uninfected and *Bb*-infected mice at weeks 2 and 4 post-infection. Circles show regions of interest (ROIs) selected for spatial transcriptomics, each circle contained approximated 100 cells. Tissue sections were stained with SYTO-13 (blue; nucleic acid counterstain), anti-I-$E^k$ (red; MHC class II marker), and anti-Vimentin (VIM; yellow). Week 2 Uninfected, Week 2 Infected, Week 4 Uninfected, and Week 4 Infected are shown. (B) Table with probe numbers for each tissue.

Gura et al,
Supplementary Figure 2

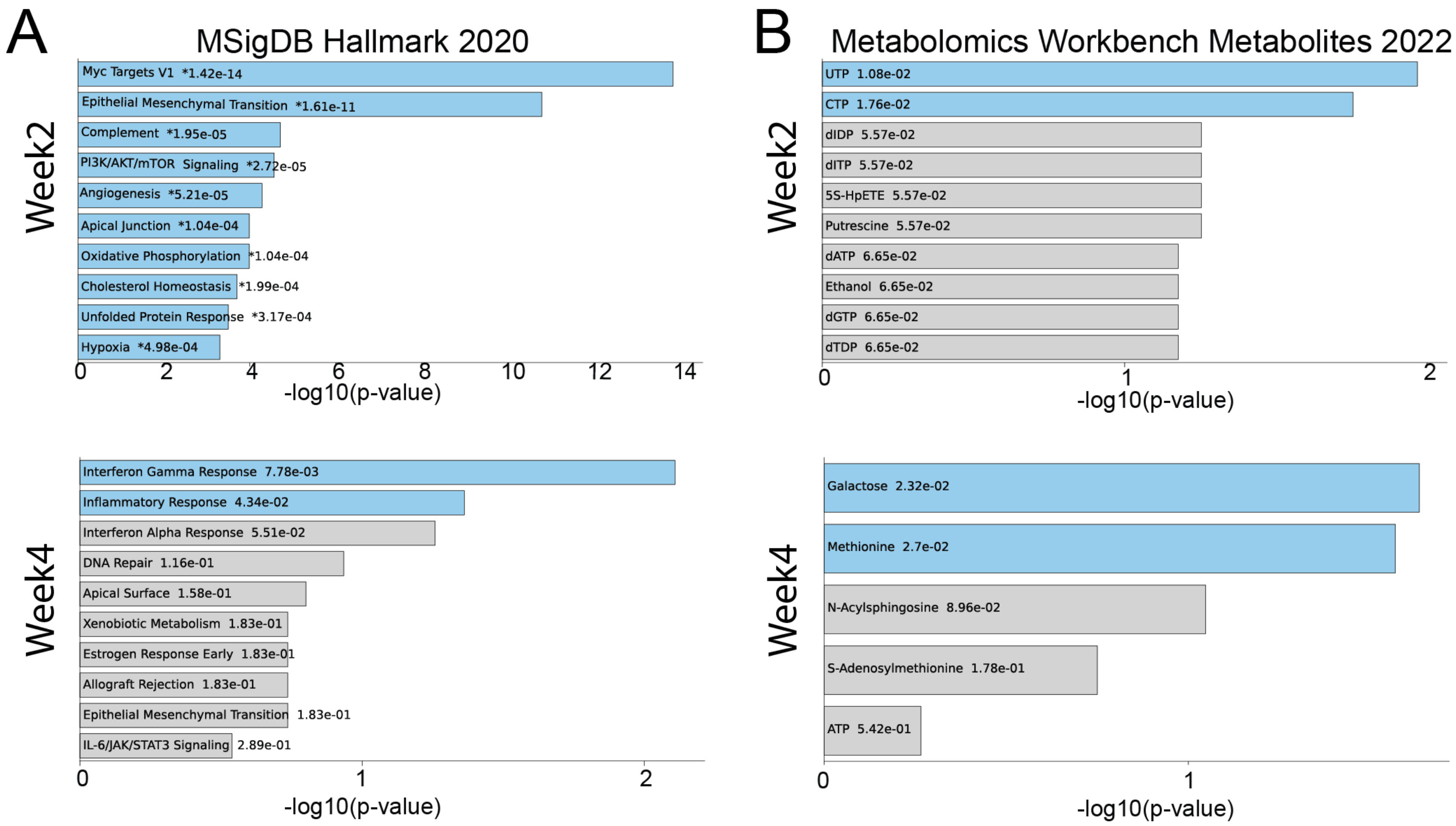


bioRxiv preprint doi: https://doi.org/10.64898/2026.06.25.734510; this version posted June 25, 2026. The copyright holder for this preprint (which was not certified by peer review) is the author/funder, who has granted bioRxiv a license to display the preprint in perpetuity. It is made available under a CC-BY 4.0 International license.

bioRxiv preprint doi: https://doi.org/10.64898/2026.06.25.734510; this version posted June 25, 2026. The copyright holder for this preprint (which was not certified by peer review) is the author/funder, who has granted bioRxiv a license to display the preprint in perpetuity. It is made available under a CC-BY 4.0 International license.

**Supplementary Figure 2.** Pathway analysis for downregulated genes following 2 and 4 weeks of *Bb* infection. Top enriched MSigDB Hallmark pathways (A) and Metabolomics metabolites (B) in the downregulated genes at week 2 (top two graphs) and week 4 (bottom two graphs) after *Borrelia* infection. X axis indicates –log10(p-value) respectively were determined using EnrichR and Appyter. Blue bars are more significant, while gray bars have less significance.

bioRxiv preprint doi: https://doi.org/10.64898/2026.06.25.734510; this version posted June 25, 2026. The copyright holder for this preprint (which was not certified by peer review) is the author/funder, who has granted bioRxiv a license to display the preprint in perpetuity. It is made available under a CC-BY 4.0 International license.

Gura et al,
Supplementary Figure 3

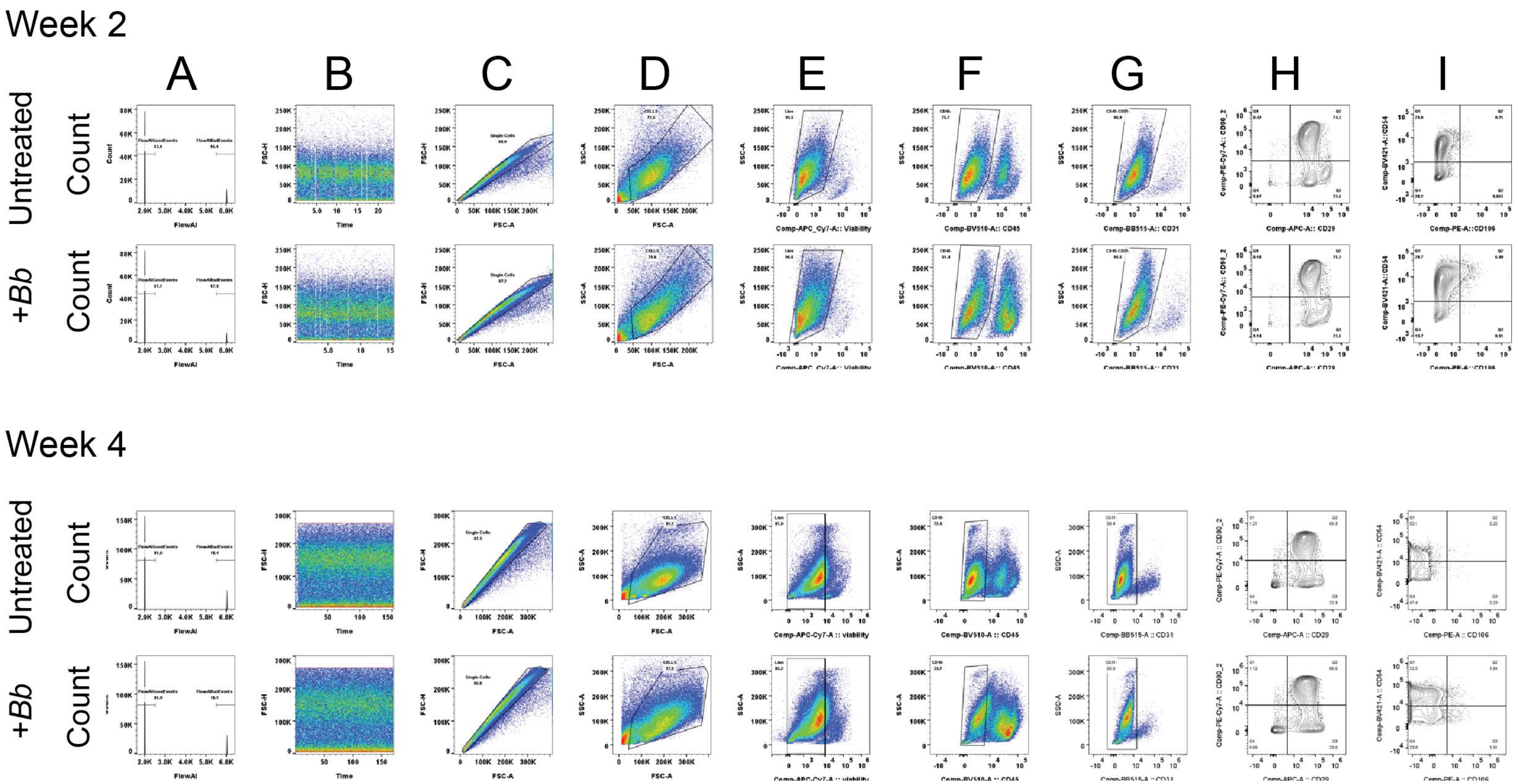

bioRxiv preprint doi: https://doi.org/10.64898/2026.06.25.734510; this version posted June 25, 2026. The copyright holder for this preprint (which was not certified by peer review) is the author/funder, who has granted bioRxiv a license to display the preprint in perpetuity. It is made available under a CC-BY 4.0 International license.

**Supplementary Figure 3. Gating strategy.**

Unstained controls were used to set gates for each fluorochrome, and single-stained controls were used to calculate compensation. The hierarchical gating strategy for the identification of double-positive cells expressing the adhesion molecules CD106 (VCAM-1) and CD54 (ICAM-1) within the CD29+CD90.2+ synovial stromal cell population is illustrated with representative plots from VEHICLE and Borrelia groups at week 2 and week 4 post infection (panels A-I).

At least 100,000 were collected per sample. Prior to downstream gating, data quality was assessed using the FlowAI plugin (FlowJo v10.10.1) to automatically detect and quantify anomalous events based on default quality control metrics (flow rate, signal acquisition and dynamic range). A representative histogram (A) and time (x-axis) versus forward scatter-height (FSC-H, y-axis) plot (B) illustrate the distribution of "good events" (retained for further analysis) and bad events (flagged/anomalous) events. Singlets were then gated from good events on an FSC-area (FSC-A) versus FSC-H plot to exclude doublets and aggregates (C). The singlet population was then gated on FSC-A versus side scatter-area (SSC-A) to remove residual debris and select cells for further analysis (D). Viable cells were selected as cells negative for the viability dye (Fixable Viability Dye APC-Cy7) within the FSC-A/SSC-A gated cells (E). CD45 expression versus SSC-A on live cells was used to discriminate against hematopoietic lineages (CD45+) populations (F). The CD45- subpopulation was gated for further gating of CD31- events to remove endothelial contaminants (G). Synovial stromal cells were identified as CD29 (Integrin-b1) and CD90.2 (Thy-1.2) double-positive cells within CD45-CD31- population (H). Cells double positive for the adhesion molecules CD106 (VCAM-1) and CD54 (ICAM-1) were identified within the CD29+CD90.2+ synovial stromal cell gate (I).

bioRxiv preprint doi: https://doi.org/10.64898/2026.06.25.734510; this version posted June 25, 2026. The copyright holder for this preprint (which was not certified by peer review) is the author/funder, who has granted bioRxiv a license to display the preprint in perpetuity. It is made available under a CC-BY 4.0 International license.

Gura et al,
Supplementary Figure 4

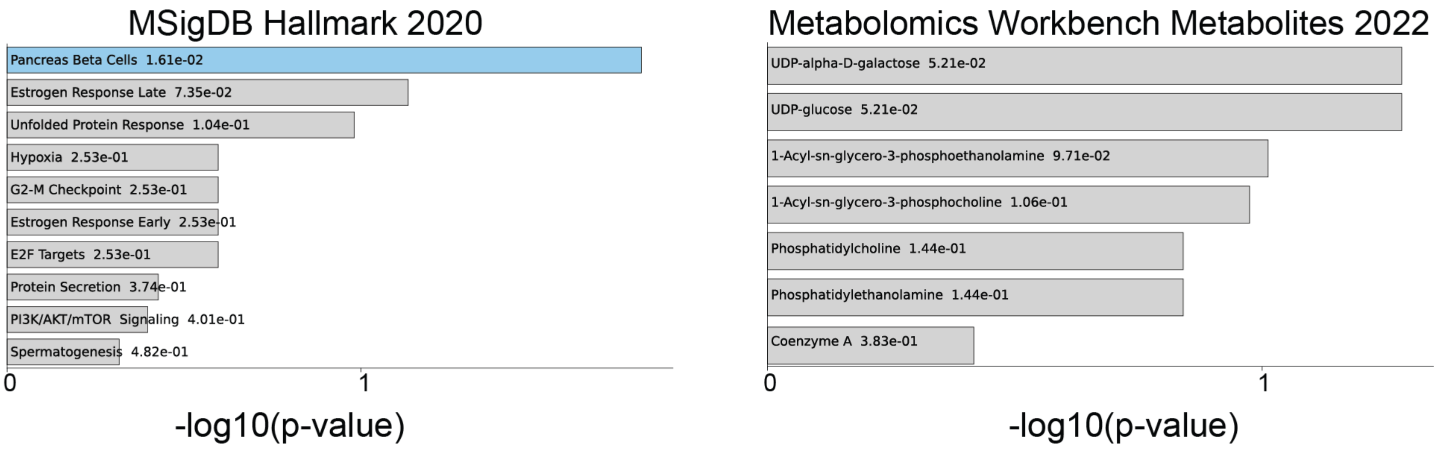

bioRxiv preprint doi: https://doi.org/10.64898/2026.06.25.734510; this version posted June 25, 2026. The copyright holder for this preprint (which was not certified by peer review) is the author/funder, who has granted bioRxiv a license to display the preprint in perpetuity. It is made available under a CC-BY 4.0 International license.

**Supplementary Figure 4. Pathway analysis for upregulated genes in lateral synovium after 2 weeks of *Bb* infection.** Top enriched MSigDB Hallmark pathways (A) and Metabolomics metabolites (B) in the upregulated genes at week 2 after *Borrelia* infection. X axis indicates –log10(p-value) respectively were determined using EnrichR and Appyter. Blue bars are more significant, while gray bars have less significance.
